# Immune classification of advanced melanoma identifies non-responders to anti-PD1 therapy

**DOI:** 10.1007/s00262-026-04392-1

**Published:** 2026-04-28

**Authors:** Angelo Gámez-Pozo, Lucía Trilla-Fuertes, Fernando Becerril-Gómez, Pedro Lalanda-Delgado, Virtudes Soriano, Fernando Garicano Goldaraz, M. José Lecumberri, María Rodríguez de la Borbolla, Margarita Majem, Elisabeth Pérez-Ruiz, María González-Cao, Juana Oramas, Rocío López-Vacas, Alejandra Magdaleno, Joaquín Fra, Alfonso Martín-Carnicero, Mónica Corral, Teresa Puértolas, Ricardo Ramos-Ruiz, Enrique Espinosa, Juan Ángel Fresno Vara

**Affiliations:** 1 Molecular Oncology Lab, Institute of Medical and Molecular Genetics-INGEMM, Hospital Universitario La Paz-IdiPAZ, Madrid, Spain; 2 Instituto Valenciano de Oncología, Valencia, Spain; 3 Oncology, Biobizkaia Health Research Institute, Galdakao Usansolo Hospital, Osakidetza, Galdakao, Bizkaia, Spain; 4 Complejo Hospitalario de Navarra, Pamplona, Spain; 5 Hospital de Valme, Seville, Spain; 6 Hospital de la Santa Creu i Sant Pau, Barcelona, Spain; 7 Unidad de Gestión Clínica Intercentros (UGCI) de Oncología Médica, Hospitales Universitarios Regional y Virgen de la Victoria, Instituto de Investigación Biomédica de Málaga (IBIMA), Hospitales Universitarios Regional y Virgen de la Victoria, Málaga, Spain; 8 Hospital Quirón Dexeus, Barcelona, Spain; 9 Hospital Universitario de Canarias-San Cristóbal de la Laguna, Santa Cruz de Tenerife, Tenerife Spain; 10 Hospital Universitario de Elche y Vega Baja, Alicante, Spain; 11 Hospital Universitario Río Hortega, Valladolid, Spain; 12 Hospital San Pedro, Logroño, Spain; 13 Hospital Clínico Lozano Blesa, Zaragoza, Spain; 14 Hospital Universitario Miguel Servet, Zaragoza, Spain; 15 IMDEA Nutrition | IMDEA Food ES, Madrid, Spain; 16 Medicine Department, Universidad Autónoma de Madrid, Madrid, Spain; 17 Medical Oncology Service, Hospital Universitario La Paz, Madrid, Spain; 18 Biomedical Research Networking Center on Oncology-CIBERONC, ISCIII, Madrid, Spain

**Keywords:** Melanoma, Immunotherapy, Immune signature, Response to treatment

## Abstract

**Background:**

Immunotherapy based on anti-PD1 inhibitors has significantly improved survival in advanced melanoma. However, a significant proportion of patients do not benefit, and predicting response to immunotherapy remains an area of unmet need. Our group previously defined an immune signature able to predict response to anti-PD1 inhibitors in this scenario.

**Methods:**

In this study, we analyzed two cohorts of patients with advanced melanoma treated with anti-PD1 inhibitors: the GEM cohort, previously used to validate our immune signature, and Campbell’s cohort, which contains data about different immunotherapy schemes. Using the 107 genes that compose our immune signature and consensus clustering, samples were classified as immune-low or immune-high. Then, CIBERSORTx and Ecotyper were used to estimate the proportion of each immune cell type and carcinoma ecotypes in both cohorts.

**Results:**

We confirmed that the immune-low group includes mostly patients who do not response to anti-PD1 inhibitors. We also studied the distribution of carcinoma ecotypes in the immune-high and immune-low groups defined by our immune classification. Ecotypes CE9 and CE10 clustered in the immune-high group, with good response to treatment. The use of combination immunotherapy improved response rate both in immune-low and immune-high tumors. The immune-high group contained a higher number of CD8 T cells, B memory cells and T follicular helper cells.

**Conclusions:**

Our immune-based classification defines an immune-low group of tumors with poor response to anti-PD1 inhibitors. This immune classification is related to carcinoma ecotypes. Finally, a use of a combo scheme improves the rates of response both in immune-high and low groups but in the case of immune-low tumors, our results suggests that a combo treatment approach could be an adequate strategy and should be further explored in these patients. Altogether, our results support the utility of our immune signature in the prediction of response to anti-PD1 inhibitors in advanced melanoma.

**Supplementary Information:**

The online version contains supplementary material available at 10.1007/s00262-026-04392-1.

## Introduction

Immunotherapy is one of the cornerstones of systemic therapy for advanced melanoma. Anti-PD1 antibodies, either alone or in combination with anti-CTLA4 or anti-LAG antibodies, obtain long-term survival in over 40% of patients with this condition. Unfortunately, one third of the patients have a progression in the first six months of therapy and an additional 20–30% will progress thereafter. For this reason, predictive biomarkers are needed [1].

Our group previously defined an immune signature in the TCGA cohort, which included patients not treated with immunotherapy. This classification identifies two groups of patients with different immune status but has no prognostic value. However, when this classification was applied to a cohort treated with anti-PD1 inhibitors, it identified a group of patients with better overall survival, which suggests a treatment response predictive value [2].

Additionally, it is well-known that immune populations present in the tumor microenvironment have a crucial role in response to immunotherapy. Tools based on deconvolution analyses, such as Ecotyper and CIBERSORT [3, 4], allow the estimation of immune populations in a sample from bulk RNA-seq data, facilitating the understanding of tumor–immune system interactions and their impact in clinical outcomes.

In this work, we combined our previously defined immune signature with both CIBERSORTand Ecotyper to characterize response to immunotherapy in advanced melanoma.

## Material and methods

Data from two different cohorts of patients with advanced melanoma treated with anti-PD1 inhibitors were used. The Spanish Melanoma Group (GEM) cohort had been previously used to define our immune signature. The cohort in the study by Campbell et al. was used as an additional validation. In this cohort, anti-PD1 pre-treatment samples from cutaneous melanoma were selected. Complete and partial responses were considered as a good of favorable response.

### Spanish melanoma group (GEM) cohort

Fifty-two formalin-fixed paraffin-embedded (FFPE) pre-treated samples from advanced melanoma patients treated with anti-PD1 inhibitors pembrolizumab or nivolumab were recruited by the Spanish Melanoma Group (GEM). This cohort has been used in other two previous studies [2, 5]. RNA was isolated from these samples and RNA-seq sequencing experiments were performed.

The inclusion criteria were: cutaneous melanoma, treatment with either pembrolizumab or nivolumab, formalin-fixed paraffin-embedded (FFPE) sample available. Approval from the Ethics Committee of Comunidad Foral de Navarra was obtained (EO17/23) and written informed consent for each participant in the study was collected.

### Campbell et al. cohort

The RNA-seq data and the corresponding metadata analyzed in the study done by Campbell et al. [6] were downloaded from GitHub (https://github.com/ParkerICI/MORRISON-1-public). From this dataset, only data from patients with cutaneous melanoma, treated with either pembrolizumab or nivolumab, and pre-PD1 treatment samples were selected. Information about response to PD1 inhibitors is available but no info about OS is. Cohorts were analyzed separately according to treatment in three groups: no prior anti-CTLA4 treatment before sample obtaining (ipi-naïve), prior anti-CTLA4 before sample obtaining (ipi-experienced), and after sample obtaining treatment with anti-PD1 plus anti-CTLA4 (Combo).

### RNA isolation from GEM samples

Five to ten 10 µm sections from each formalin-fixed paraffin-embedded (FFPE) sample of the GEM cohort was used for RNA extraction. RNA was isolated using miRNeasy FFPE kit (QIAGEN), following manufacturer’s instructions. Obtained RNA was qualified and quantified in a NanoDrop spectrophotometer (Thermo Fisher).

### RNA-seq sequencing and RNA-seq data preprocessing from GEM samples

Genes involved in immune processes were selected for these experiments. 100 ng of RNA per sample were used for library preparation with KAPA RNA Hyperprep kit (Roche Nimblegen Inc.). Library fragment distribution was checked by electrophoresis and concentration was measured using APA library Quantification kit (Roche Nimblegen Inc.). A seven MB SeqCap EZ probe pool (Roche), including the genes previously selected, was designed using the NimbleDesign online tool. Then, an equal mass of eight cDNA libraries was pooled and hybridized using the SeqCap EZ probe pool. Samples were sequenced using paired end 2 × 100 NextSeq 50/550 high Output Cartridge v2, 75 cycles. Mapping against human genome and FPKM calculation were performed using TopHat and Cufflink, both included in GPRO Suite Tool (Biotechvana) [7].

Ensembl gene notation from sequencing raw data was translated to official gene symbol using Biomart tool [8]. After that, seven gene symbols were duplicated so the FPKMs of these genes were added to each other. Then, a filter consisting of genes with at least 400 counts in all the analyzed samples were applied. Data was log2 transformed and an extra filter consisting of genes with at least 50% of non-zero values were applied. Finally, missing values were imputed following an normal distribution using Perseus [9]. Raw RNA-seq data are publicly available at https://www.ebi.ac.uk/biostudies/arrayexpress under the identifier E-MTAB-11729.

### Assignation to immune groups

Assignation to previous defined immune groups was done using Consensus Clustering (CC). The expression of one hundred and seven genes defined in melanoma TCGA cohort as our immune classification was used to perform the CC analysis [2]. CC was done using ConsensusClusterPlus R package v1.72.0 [10].

### Use of deconvolution methods to estimate the immune cell proportions in each sample and the carcinoma ecotypes

CIBERSORTx [3] and Ecotyper [4] were used to estimate the proportion of each immune cell type and carcinoma ecotypes (CEs). Ecotyper provides information about immune configuration of the sample whereas CIBERSORT estimated the immune cell populations present in a sample from bulk RNA-seq data. CIBERSORT and Ecotyper were run independently for each cohort. In the case of CIBERSORT, no absolute mode or permutations were used.

### Statistical analyses

For comparisons between groups, nonparametric Mann–Whitney or Kruskal–Wallis with Dunn test were applied. P values were adjusted for multiple testing by FDR. In order to compare distributions of ecotypes according to immune signature groups or response to anti-PD-1 inhibitors, Chi-squared test, applying Yates correction or Fisher’s exact test when it is necessary, was calculated. Parameters of survival analyses were calculated using Kaplan–Meier and Cox regression. All p values were bilateral and considered statistically significant under 0.05. These analyses were performed in GraphPad Prism version 6.

## Results

### Clinical characteristics of the Spanish melanoma group (GEM) cohort

The cohort from the Spanish Melanoma Group (GEM) has been previously analyzed [2, 5]. This cohort includes samples from 52 patients with melanoma in stage IV treated with anti-PD1 inhibitors (samples included 26 primary tumors, 10 metastatic lymph nodes, and 16 distant metastases). Median of follow-up was 12.6 months. Eleven patients had a complete response, 13 a partial response, 10 stable disease, and 13 progressive disease as best response. In the other five patients the response to anti-PD1 inhibitors was not evaluable. Sup Table 1 summarizes the characteristics of this cohort.

### *Clinical characteristics of *Campbell cohort

Non-cutaneous melanomas were excluded in this cohort, which left 116 patients treated with anti-PD1 and not anti-CTLA4 (ipi-naïve), 48 with previous anti-CTLA4 (ipi-experienced), and 30 with the combination of anti-PD1 plus anti-CTLA4 inhibitors (combo). The clinical characteristics of both cohorts are summarized in Sup Table 2.

### RNA-seq experiments in GEM cohort

Fifty-two FFPE samples were collected for this study. However, four of them did not have enough material to perform RNA extraction. After RNA extraction, another eight samples were excluded because they had a low quantity of RNA. Finally, forty samples were analyzed by RNA-seq. Applying the two filtering criteria mentioned in methods section, 2.151 genes remained for the subsequent analyses.

### Immune classification in both cohorts

**Fig. 1:**
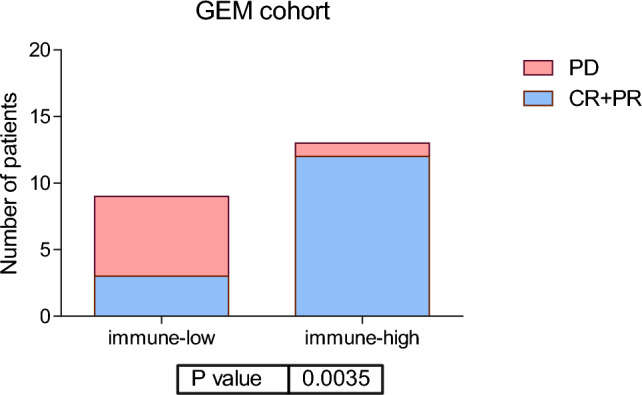
Percentage of response (CR + PR) and no response (PD) in the immune groups in GEM cohort

In a previous study, we defined an immune signature in melanoma that identifies good responders to anti-PD1 inhibitors. This signature divided samples from the GEM cohort into immune-low and immune-high groups, and the immune-high group had a favorable response to anti-PD1 therapy (Fig. 1).

Samples in the Campbell’s cohort were classified according to this immune signature as well. Samples were classified into two groups, one hundred and one (61%) samples were classified in a group with a higher expression of immune genes, the immune-high group, and sixty-four (39%) were classified in a group that showed a lower expression of these immune genes, the immune-low group. The mean expression of the immune genes in each defined groups is shown in Sup Fig. 1 and Sup Table 3.

**Fig. 2:**
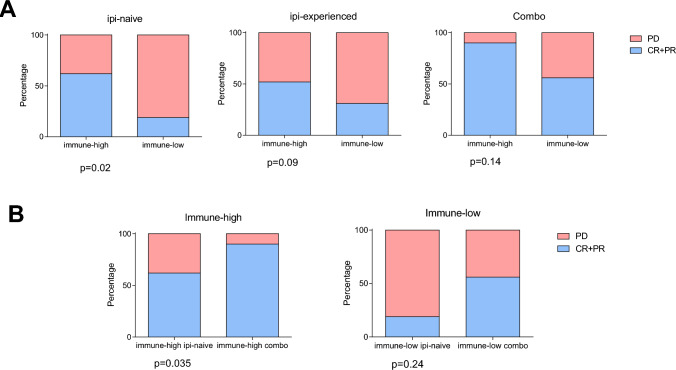
A. Percentage of response (CR + PR) and no response (PD) in the immune groups in Campbell cohort separated by treatment. B. Percentage of response (CR + PR) and no response (PD) in the immune groups comparing ipi-naïve and combo in Campbell cohort

The immune-high group had a higher response, regardless of the treatment (difference statistically significant in the ipi-naïve population) (Fig. 2A). Response rate was similar in the ipi-naïve and ipi-experienced patients within this immune-high group (Fig. 2A). Combination immunotherapy improved the rate of responses both in immune-low and immune-high tumors, being statistically significant in the immune-high group (Fig. 2B).

### Distribution of carcinoma ecotypes according to the groups defined by our immune signature

**Fig. 3:**
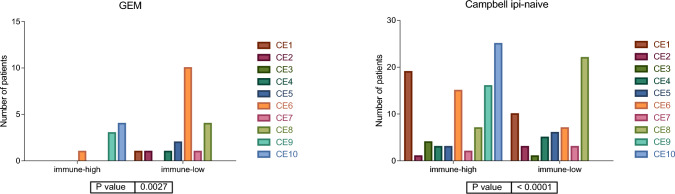
Distribution of the ecotypes in the immune groups in GEM and Campbell cohorts. Distribution of the carcinoma ecotypes according to treatment history

Carcinoma ecotypes (CEs) defined by Ecotyper consist on distinct cell-state co-occurrence patterns that correlate with cancer progression and treatment response. CE1 and CE2 are immunologically cold. CE3 is enriched with immunosuppressive myeloid cells. CE4 had a depletion of B cell receptor signaling. CE5 and CE6 showed an intermediate profile. CE7 and CE8 had a moderate immune infiltration and CE9 and CE10 were enriched in CD8 + T cells [4, 11].We studied the distribution of CEs in the immune-high and immune-low groups defined by our immune signature. The distribution of CE in immune-high and low groups was significantly different, being all CE9 and CE10 located in the immune-high group (Fig. 3).

**Fig. 4:**
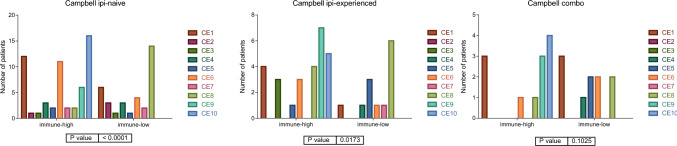
Distribution of the ecotypes in Campbell cohort according to treatment history

Differences in ecotype distribution in immune groups has remained independently of anti-CTLA4 prior administration in Campbell’s cohort. No statistical difference was found in the combo group, probably due to the reduced number of samples in this group (Fig. 4).

### Distribution of the carcinoma ecotypes according to response to treatment

**Fig. 5:**
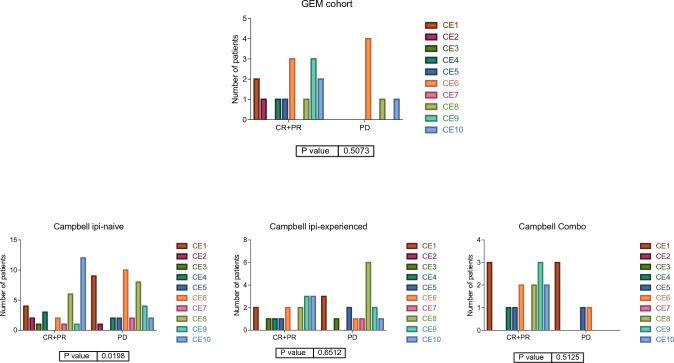
Distribution of ecotypes in responders (CR and PR) and non-responders (PD) to treatment in GEM and Campbell cohorts

We also checked the ecotype distribution according to response to anti-PD1 inhibitors. GEM cohort was treated with anti-PD1 inhibitors, either with or without ipilimumab. Campbell’s cohort was analyzed according to therapy: ipi-naïve, ipi-experienced and combo. Distribution of ecotypes according to response to anti-PD1 inhibitors were differential in the case of ipi-naïve patients (*p* = 0.019), i.e., the ecotype correlated with response to anti-PD1 inhibitors only if the patient had not previously received an anti-CTLA4 antibody. In the case of the Combo, no definitive conclusions could be achieved, probably due to the reduced number of samples assigned by Ecotyper, but a trend that CE9 and CE10 were related to a good response to treatment was observed (Fig. 5).

### Differential immune cell populations in immune groups according to CIBERSORT predictions

CIBERSORT was used to estimate the immune populations present in each immune group. The immune-high group showed higher levels of plasma cells, T CD8 cells, T CD4 memory activated cells, T follicular helper and macrophages M1 than the immune-low group in both GEM and Campbell’s cohorts (Sup Fig. 2).

**Fig. 6:**
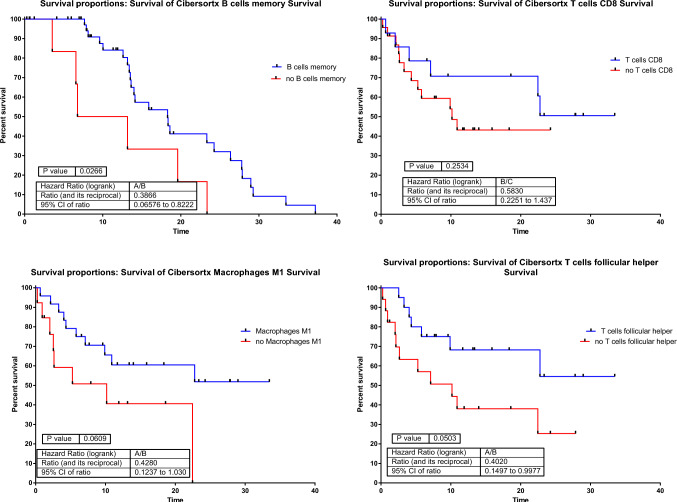
Cell populations whose presence is related to overall survival in GEM cohort

Regarding the presence of immune cells related to OS in the GEM cohort, B cells memory, CD8 T cells, and T cells follicular helper presence were correlated with better overall survival (Fig. 6).

## Discussion

Immunotherapy has significantly improved the outcome of patients with advanced melanoma. However, not all patients respond to this treatment [1]. We previously defined an immune signature that identifies patients with a favorable response to anti-PD1 therapy [2]. In the present study, deconvolution methods were used to further characterize the immune configuration and immune cells involved in this good response [3, 4].

We confirmed that objective responses were more common in the immune-high group, regardless of the treatment administered (Fig. 2A). Prior exposure to ipilimumab did not modify the result in either the immune-high or the immune-low groups. The lack of effect of previous exposure to anti-CTL4 has been found in other studies [12]. On the contrary, the Combo improved response rate both in immune-low and immune-high tumors, being statistically significant in the immune-high group (Fig. 2B). This suggests that patients with immune-low tumors may do not benefit from single-agent anti-PD1 therapy and should rather be treated with combination immunotherapy. Patients with immune-high tumors seem also to derive more benefit from the combination, but they have a good response rate with single-agent therapy, so this would be an acceptable choice (Fig. 2). These findings are in line with the DOMINI results, where patients with a high interferon signature had a better response to anti-PD1 monotherapy, whereas patients with a low interferon signature did better with the combination of anti-PD1 plus anti-CTLA4 [13]. Indeed, four genes overlaps between the two signatures: CXCL9, CXCL10, CXCL11, and GZMA [14].

Regarding the ecotypes, the immune-high group contained CE9 and CE10. Both ecotypes were previously correlated with a favorable response to immunotherapy in the Ecotyper study [4].

CE9 is characterized by CD4 T cells S01 (Exhausted/effector memory/Treg), CD8 T cells S03 (Exhausted/effector memory), B cells S05 (activated), dendritic cells S03 (mature, immunogenic), macrophages S03 (M1), NK cells S01 (classical) and plasma cells S02 (unknown). Relative abundance of partially exhausted CD8 T cells predicts response to anti-PD1 therapy [15]. Macrophages M1 and classical NK cells have been associated with favorable outcomes in other tumor types. Macrophages M1 and NK cells S01 has also been associated with better response to immunotherapy [4]. CE10 is characterized by CD8 T cells S01 (Naïve/central memory), B cells S01 (Classical naïve), CD4 T cells S02 (Naïve/central memory), dendritic cells S01 (myeloid cDC1), mast cells S04 (classical), macrophages S01 (monocytes), and plasma cells S01 (classical) [4]. Naïve central memory T cells, cDC1 myeloid dendritic cells, and classical plasma cells were associated with favorable outcomes across carcinomas [4], and they are involved in CD8 T cell activation [16].

To confirm the utility of our immune classification, we also studied the distribution of ecotypes according to response to anti-PD1 inhibitors. In this case, differences in the response rate in ecotypes were only found in ipi-naïve patients, i.e., it seems that the ecotype is related to response to anti-PD1 inhibitors when the patient has not previously received anti-CTLA4 treatment but not if the patient has previously received it. In the case of Combo, no definitive conclusions can be achieved probably due to the reduced number of samples assigned by Ecotyper, but a trend that CE9 and CE10 correlate with a good response to treatment was observed. On the contrary, when our immune signature was applied, differences in the distribution of responses according to the immune groups can be found in all treatment schemes, having the immune-low group the lower response rate in all cases. Ecotypes have been defined as set of genes characteristic of ten different immune configurations. Our immune signature suggests that in advanced melanoma treated with PD1 scenario, it is possible to simplify these configurations into two states, immune-low and immune-high.

CIBERSORT was used to characterize the immune populations present in each immune group. Immune-high group showed a higher proportion of CD8 T cells, CD4 T memory activated cells, T follicular helper and macrophages M1 than immune-low group in both GEM and Campbell’s cohorts. The presence of effector T cells is essential for the benefit from immune checkpoint blockade inhibitors, especially T CD8 [17]. Higher number of CD8 T cells in pre-treated samples was associated with response to pembrolizumab and in serially tumor samples, the amount of CD8 T cells positively correlates with reduction in tumor size [18]. Single-cell studies have shown that CD8 T cells, B cells and CD4 memory activated T cells are enriched in melanomas with a good response to ICIs [19]. Therefore, it is not surprising that these immune cells are present in the immune-high group. In addition, the presence of these immune cell populations is related to a better OS in the GEM cohort, confirming not only their relationship with a better response to anti-PD1 but also with survival.

Our study has some limitations. Both series of patients showed a correlation of the immune signature with response, but the Campbell’s cohort does not provide information on overall survival, not being possible validating also the prognostic capability of our immune signature. Therefore, the validation of our immune signature in other cohorts treated with anti-PD1 inhibitors would be interesting. Moreover, the reduced number of samples included in the Combo group of Campbell’s cohort make it necessary to be cautious about drawing conclusions regarding the comparative effectiveness of treatment strategies. In addition, CIBERSORT and Carcinoma Ecotyper are widely tested to predict immune cell populations but single-cell RNA-seq experiments have probably more resolution, despite its technical difficulty and high-cost. Finally, it would be interesting to assess biopsies from patients treated with different immunotherapy schemes to analyze them by RNA-seq and validate these findings. To implement this signature in clinical practice, first a retrospective independent validation in a larger cohort is needed. Then, a prospective validation should be performed. Also, exploring a clinical-friendly platform (such as a reduced, qPCR-based signature) could help implementation in the future.

In conclusion, in this study we applied a previously defined immune signature to show that patients with immune-low advanced melanoma respond poorly to single-agent anti-PD1 inhibitors. This immune classification is related to carcinoma ecotypes. In addition, the immune-low group, show a lower response rate to monotherapy with anti-PD1 inhibitors, suggesting that a combo with other drugs should be explored as a treatment strategy in this group. Finally, CIBERSORT was used to characterize immune cell populations and the group of good responder patients present higher levels of CD8 T cells, B cell memory, macrophages M1, and T cells follicular helpers. Altogether, these results support the utility of our immune signature in the prediction of response to anti-PD1 inhibitors in advanced melanoma.

## Supplementary Information

Below is the link to the electronic supplementary material.

Supplementary file1 (DOCX 12 KB)

Supplementary file2 (DOC 35 KB)

Supplementary file3 (XLS 212 KB)

Supplementary file4 (DOCX 466 KB)

## Data Availability

RNA-seq data is publicly available at https://www.ebi.ac.uk/arrayexpress/ under the identification E-MTAB-11729.
