# Supplementary material for: Immune classification of advanced melanoma identifies non-responders to anti-PD1 therapy
Source: Cancer Immunol Immunother. 2026 Apr 28;75(5):156. doi: 10.1007/s00262-026-04392-1 (PMC13125473; doi:10.1007/s00262-026-04392-1)
Supplement: Supplementary file 1 — Supplementary file1 (DOCX 12 KB) [file 262_2026_4392_MOESM1_ESM.docx]

| **GEM cohort** | **N= 52 (100%)** |
| --- | --- |
| Age at diagnosis (median and range) | 66 (33-88) |
| Gender |  |
| Male | 35 (67.3%) |
| Female | 17 (32.6%) |
| BRAF mutation |  |
| Positive | 15 (28.8%) |
| Negative | 29 (55.8%) |
| Unknown | 8 (15.4%) |
| Anti-PD1 inhibitor |  |
| Pembrolizumab | 27 (52%) |
| Nivolumab | 25 (48%) |
| Best response to anti-PD1 inhibitor |  |
| CR+PR | 24 (46.2%) |
| PD | 10 (19.2%) |
| SS | 5 (9.6%) |
| Toxicity to anti-PD1 treatment |  |
| Yes | 10 (19.3%) |
| No | 30 (57.7%) |
| Unknown | 12 (23%) |

Sup Table 1: Summary of number and percentage of clinical characteristics of GEM cohort.
