# Supplementary material for: Immune classification of advanced melanoma identifies non-responders to anti-PD1 therapy
Source: Cancer Immunol Immunother. 2026 Apr 28;75(5):156. doi: 10.1007/s00262-026-04392-1 (PMC13125473; doi:10.1007/s00262-026-04392-1)
Supplement: Supplementary file 2 — Supplementary file2 (DOC 35 KB) [file 262_2026_4392_MOESM2_ESM.doc]

| **Campbell cohort** | **Ipi-naïve n=116 (100%)** | **Ipi-experienced n=48 (100%)** | **Combo n=30 (100%)** |
| --- | --- | --- | --- |
| Age median (range) | 61 (37-90) | 52 (44-71) | 59 (34-82) |
| Gender |  |  |  |
| Male | 68 (59%) | 26 (54%) | 20 (67%) |
| Female | 48 (41%) | 20 (42%) | 10 (33%) |
| Unknown | 0 (0%) | 2 (4%) | 0 (0%) |
| Best response to anti-PD1 |  |  |  |
| CR/PR | 48 (41%) | 17 (35%) | 20 (67%) |
| SD | 22 (19%) | 10 (21%) | 3 (10%) |
| PD | 44 (38%) | 21 (44%) | 7 (23%) |
| Unknown | 2 (2%) | 0 (0%) | 0 (0%) |

Sup Table 2: Summary of number and percentage of clinical characteristics in Campbell et al. cohort sorted by type of treatment. CR/PR: complete/partial response. SD: stable disease. PD: progressive disease.
