# Supplementary material for: Immune classification of advanced melanoma identifies non-responders to anti-PD1 therapy
Source: Cancer Immunol Immunother. 2026 Apr 28;75(5):156. doi: 10.1007/s00262-026-04392-1 (PMC13125473; doi:10.1007/s00262-026-04392-1)
Supplement: Supplementary file 4 — Supplementary file4 (DOCX 466 KB) [file 262_2026_4392_MOESM4_ESM.docx]

Sup Fig 1: Mean expression or activity of immune genes that composed our immune classification in the Campbell cohort.

Sup Fig 2: Differential immune cell abundances between the immune-groups in GEM and Campbell cohorts.
